# Concomitant Ro/SSA and La/SSB antibodies are biomarkers for the risk of venous thromboembolism and cerebral infarction in primary Sjögren's syndrome

**DOI:** 10.1111/joim.12941

**Published:** 2019-06-17

**Authors:** J. Mofors, M. Holmqvist, L. Westermark, A. Björk, M. Kvarnström, H. Forsblad‐d'Elia, S. Magnusson Bucher, P. Eriksson, E. Theander, T. Mandl, M. Wahren‐Herlenius, G. Nordmark

**Affiliations:** ^1^ Division of Rheumatology Department of Medicine Karolinska Institutet Karolinska University Hospital Stockholm Sweden; ^2^ Division of Clinical Epidemiology Department of Medicine Karolinska Institutet Stockholm Sweden; ^3^ Department of Medical Sciences Rheumatology and Science for Life Laboratory Uppsala University Uppsala Sweden; ^4^ Department of Public Health and Clinical Medicine, Rheumatology Umeå University Umeå Sweden; ^5^ Department of Rheumatology Faculty of Medicine and Health Örebro University Örebro Sweden; ^6^ Division of Rheumatology Department of Clinical Experimental Medicine Linköping University Linköping Sweden; ^7^ Department of Clinical Sciences, Malmö, Rheumatology Lund University Malmö Sweden

**Keywords:** autoantibodies, cardiovascular disease, La/SSB, Primary Sjögren's syndrome, Ro/SSA

## Abstract

**Background:**

To assess the risk of incident cardiovascular disease in patients with primary Sjögren's syndrome, overall and stratified by Ro/SSA and La/SSB autoantibody status.

**Methods:**

A cohort of patients with primary Sjögren's syndrome in Sweden (*n* = 960) and matched controls from the general population (*n* = 9035) were included, and data extracted from the National Patient Register to identify events of myocardial infarction, cerebral infarction and venous thromboembolism. Hazard ratios were estimated using cox proportional hazard regressions.

**Results:**

During a median follow‐up of 9.5 years, the overall hazard ratio (HR) was 1.6 (95% CI 1.2–2.1) for myocardial infarction, 1.2 (95% CI 0.9–1.7) for cerebral infarction and 2.1 (95% CI 1.6–2.9) for venous thromboembolism. Patients positive for both Ro/SSA and La/SSB autoantibodies had a substantially higher risk of cerebral infarction (HR 1.7, 95% CI 1.0–2.9) and venous thromboembolism (HR 3.1, 95% CI 1.9–4.8) than the general population. These risks were not significantly increased in Ro/SSA‐ and La/SSB‐negative patients. Among autoantibody‐positive patients, the highest HR of cerebral infarction was seen after ≥10 years disease duration (HR 2.8, 95% CI 1.4–5.4), while the HR for venous thromboembolism was highest 0–5 years after disease diagnosis (HR 4.7, 95% CI 2.3–9.3) and remained high throughout disease duration.

**Conclusions:**

Primary Sjögren's syndrome is associated with a markedly increased risk of cardiovascular disease and the presence of Ro/SSA and La/SSB autoantibodies identify the subgroup of patients carrying the highest risk. These findings suggest that monitoring and prevention of cardiovascular disease in this patient group should be considered.

## Graphical abstract

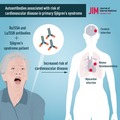

## Introduction

Primary Sjögren's syndrome is a systemic autoimmune disease, characterized by dysfunction of salivary and lacrimal glands leading to sicca symptoms 1, 2. The clinical presentation often includes arthralgia, myalgia and fatigue, and a subgroup of patients present with systemic manifestations such as cutaneous vasculitis, polyneuropathy and interstitial lung disease 3, 4, 5, 6. Serologically, autoantibodies to the Ro/SSA and La/SSB antigens can be detected 7, 8, 9. These autoantibodies can induce production of type I IFN, and associate with disease severity and systemic manifestations 10, 11, 12.

Inflammation contributes to cardiovascular disease, and increased morbidity and premature mortality related to cardiovascular events have been reported for rheumatoid arthritis (RA) and systemic lupus erythematosus (SLE) 13, 14, 15. In Sjögren's syndrome, register‐based studies have addressed the risk of cardiovascular disease, but with inconsistent results 16, 17, 18, 19, 20, 21, 22, 23. However, to the best of our knowledge, no study has previously investigated the long‐term risk of cardiovascular disease in a large cohort of clinically validated patients with primary Sjögren's syndrome. Further, the potential impact of Ro/SSA and La/SSB autoantibodies on the risk of cardiovascular disease has not been assessed in large cohorts. In the present study, we therefore investigated the incidence and relative risk of cardiovascular events (myocardial infarction, cerebral infarction and venous thromboembolism) in close to 1000 well‐characterized patients with primary Sjögren's syndrome compared with individuals from the general population with a median follow‐up period approaching 10 years, overall, and stratified by Ro/SSA and/or La/SSB autoantibody status.

## Material and methods

### Study design

We performed a cohort study of patients with primary Sjögren's syndrome included at diagnosis (incident cases) with matched general population comparators, based on prospectively recorded register data. The publicly funded Swedish healthcare system enables access to all healthcare services, including specialized care for chronic diseases, for all residents. All residents are assigned a unique personal identity number that can be used for linkage of different data resources, including several national health registers of high quality 24.

### Study population

Patients with primary Sjögren's syndrome (*n* = 960) diagnosed between 1987 and 2013 at the Departments of Rheumatology at the University Hospitals in Gothenburg, Malmö/Lund, Linköping, Örebro and Uppsala, as well as the Karolinska University Hospital in Stockholm, Sweden were included in the study. All patients received their diagnosis by a specialist in rheumatology at each centre, and all fulfilled the American–European consensus group (AECG) criteria 25. Clinical parameters related to diagnosis, including autoantibody status, were collected through patient chart review.

For each patient, ten controls from the general population (matched on sex, age and region of residency 10 years before the matched case's diagnosis date) were randomly selected from the Swedish Total Population Register at Statistics Sweden (http://www.scb.se/en). Matching prior to Sjögren's syndrome diagnosis date was chosen due to patients reporting onset of disease‐specific symptoms several years before diagnosis 5. Controls were required to be alive and resident in Sweden at the date of the matched case's diagnosis, resulting in a cohort of *n* = 9035 controls. The study was approved by the Regional Ethical Review Board in Stockholm, Sweden.

### Data sources used to detect outcomes during follow‐up

Using the national personal identification number, we linked the cohort of patients with primary Sjögren's syndrome and the matched general population comparator cohort with data from the following registers, for which data were available through 31 December 2013: The Swedish National Patient Register (NPR), the Population Register and the Cause of Death Register. The NPR has a nationwide coverage of hospitalizations (inpatient care) since 1987 and of specialist outpatient care (excluding primary care by general practitioners) since 2001. The register lists date of admission, date of discharge and the discharge diagnosis (primary and secondary diagnoses) as set by the discharging physician and classified according to the calendar year‐specific version of the International Classification of Diseases (ICD). The coverage is 99% for hospitalizations and 80% for outpatient care, with the latter lower coverage mainly due to lower reporting from private healthcare providers 26. The Population Register includes information on deaths, emigration and immigration for the entire Swedish population. The Cause of Death Register holds information on cause of death since 1962 (primary and secondary diagnosis), coded according to ICD. Through these linkages, we identified all hospitalizations and nonprimary care outpatient visits after primary Sjögren's syndrome diagnosis date, and all emigrations and deaths during follow‐up.

### Definition of outcomes and follow‐up

Using the NPR and Cause of Death Register, we assessed the risk of three types of cardiovascular disease: Myocardial infarction (MI), cerebral infarction and venous thromboembolism (VTE).

MI was defined as hospitalization listing a primary ICD‐10 diagnosis of I21 (acute myocardial infarction), or an acute myocardial infarction listed as an underlying cause of death. This definition has a positive predictive value (PPV) of 95% 27. Cerebral infarction was defined as a primary ICD‐10 diagnosis of I63 in the inpatient register, or listed as an underlying cause of death, where diagnosis validation has shown a PPV of > 95% 26. VTE was defined as a composite measure of pulmonary embolism (ICD‐10: I26) and deep vein thrombosis (DVT) (ICD‐10: I80.1‐I80.2, I81, I82.2‐I82.9). Primary and secondary diagnoses in the inpatient register and cause of death register were included; diagnoses of DVTs in outpatient care were also included. A previous study examining the validity of VTEs in the NPR suggests that this definition holds a satisfactory validity 28. For records of diagnoses predating 1997, corresponding ICD‐9 codes were used (Table S1).

The primary Sjögren's syndrome and comparison cohorts were followed from the Sjögren's syndrome diagnosis date until the first cardiovascular event, death, emigration, or 31 December 2013, whichever came first.

### Statistical analyses

The aim of this study was to investigate the risk of incident cardiovascular disease in patients with primary Sjögren's syndrome. Therefore, participants with a record of MI, cerebral infarction or VTE before the Sjögren's syndrome diagnosis date were excluded from the respective follow‐up. Differences in the proportions of individuals with prior cardiovascular disease were assessed using a conditional logistic regression conditioned on the matching factors.

Crude incidence rates are expressed as events per 1000 person‐years. Confidence intervals were estimated for incidence rates by assuming that the number of events followed a Poisson distribution. To compare the risk of incident cardiovascular disease in patients with Sjögren's syndrome with that in individuals in the comparison cohort, Cox proportional hazard models were used to estimate the hazard ratio (HR) and 95% confidence interval (CI), using time since Sjögren's syndrome diagnosis as time scale. Proportionality of the hazards was tested using scaled Schoenfeld residuals. No evidence of departure from this assumption was observed for the outcomes considered in this study. In addition, incidence and HR were assessed separately by time since Sjögren´s syndrome diagnosis (0 to <5, 5 to <10, ≥10 years), age (<50, 50 to 70, >70 years), and by the presence of Ro/SSA and La/SSB autoantibodies.

All analyses were performed using STATA/MP version 13.0 (StataCorp LP, College Station, TX, USA). Statistical significance was defined by an alpha level of 0.05.

## Results

### Demographic and clinical characteristics of the cohort

Of the included 960 patients with primary Sjögren's syndrome, 391 (41%) were seropositive for both Ro/SSA and La/SSB autoantibodies (SSA/SSB double‐positive), 278 (29%) were positive for only one of either Ro/SSA or La/SSB antibodies (SSA/SSB single‐positive), and 274 (29%) tested negative for both antibodies (SSA/SSB‐negative) (Table 1). Seventeen patients did not have any available record on autoantibody status. The mean age at diagnosis was 55 years; SSA/SSB double‐positive patients were diagnosed on average 4.8 and 7.3 years earlier in life than SSA/SSB single‐positive (*P* < 0.0001) and SSA/SSB‐negative patients (*P* < 0.0001), respectively. The median time of follow‐up (interquartile range) was 9.5 (4.5–14.6) years in the Sjögren's syndrome patient cohort and 9.5 (4.5–15.5) years in the comparison cohort.

**Table 1:** Descriptive characteristics of 960 incident cases of primary Sjögren's syndrome and 9035 matched controls from the general population

		Primary Sjögren's syndrome patients	General population controls
Size, *n* (% females)	All patients	960 (93%)	9035 (93%)
	SSA/SSB double‐positive	391 (90%)	3700 (90%)
	SSA/SSB single‐positive	278 (92%)	2595 (93%)
	SSA/SSB‐negative	274 (96%)	2583 (96%)
Age in years at primary Sjögren's syndrome diagnosis, mean (SD)	All patients	55.4 (15)	–
	SSA/SSB double‐positive	51.8* (15)	–
	SSA/SSB single‐positive	56.6** (14)	–
	SSA/SSB‐negative	59.1 (13)	–
Years of follow‐up time after primary Sjögren's syndrome diagnosis date, median (IQR)	All patients	9.5 (4.5–14.6)	9.5 (4.5–15.5)
	SSA/SSB double‐positive	9.5 (4.5–15.5)	10.5 (4.6–16.5)
	SSA/SSB single‐positive	8.9 (4.5–13.5)	9.5 (4.5–14.5)
	SSA/SSB‐negative	8.5 (3.5–14.9)	9.3 (3.5–15.5)

Seventeen primary Sjögren's syndrome patients did not have available records on Ro/SSA and La/SSB antibodies, and are hence not included in subgroup analyses.

IQR, interquartile range; SD, standard deviation; SSA, Ro/SSA antibodies; SSB, La/SSB antibodies.

Two‐sided *t*‐test for means difference: *P < 0.0001 compared to SSA/SSB single‐positive, *P* < 0.0001 compared to SSA/SSB‐negative, **P = 0.024 compared to SSA/SSB‐negative.

### Myocardial infarction

**Figure 1:**
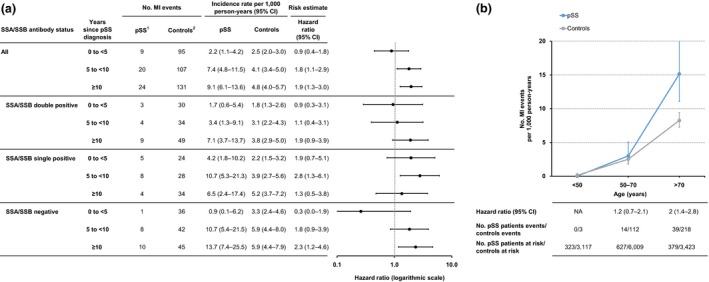
Myocardial infarction in relation to primary Sjögren's syndrome duration and age. (a) Incidence rate and hazard ratio of myocardial infarction (MI) in primary Sjögren's syndrome, stratified by years since Sjögren's syndrome diagnosis. Seventeen patients with Sjögren's syndrome did not have available records on Ro/SSA and La/SSB antibodies and are not included in subgroup analyses. (b) Age‐specific incidence rate and hazard ratio of MI with 95% confidence intervals (CI) in prevalent Sjögren's syndrome, and in population‐based comparators. ^1^Primary Sjögren's syndrome patients, ^2^General population comparators. CI, confidence interval; NA, not applicable.

The incidence rate of myocardial infarction in patients with Sjögren's syndrome was 5.6 (95% CI 4.3–7.3) per 1000 person‐years, compared to 3.6 (95% CI 3.3–4.0) in the comparison cohort, corresponding to a hazard ratio of 1.6 (95% CI 1.2–2.1) (Table 2). An increased risk was not observed during the first 5 years after diagnosis (HR 0.9, 95% CI 0.4–1.8), but was present between 5 to < 10 years (HR 1.8, 95% CI 1.1–2.9) and ≥10 years after the diagnosis of Sjögren's syndrome (HR 1.9, 95% CI 1.3–3.0) (Fig. 1a). No patient with Sjögren's syndrome had an MI before the age of 50; the incidence rate of MI was similar compared to the general population controls during the ages of 50–70 years (HR 1.2, 95% CI 0.7–2.1), but was increased after the age of 70 (HR 2.0, 95% CI 1.4–2.7) (Fig. 1b, Table S2). Female and male patients with Sjögren's syndrome presented relative risks for MI of similar amplitudes (HR 1.6, 95% CI 1.2–2.1 and HR 1.5, 95% CI 0.7–3.4, respectively), although the crude incidence rate of MI was higher in male patients (Table S3).

**Table 2:** Risk of cardiovascular events after primary Sjögren's syndrome diagnosis in an inception cohort of 960 primary Sjögren's syndrome patients and 9035 matched comparators from the general population

Event	SSA/SSB antibody status	No. events (%)		Person‐years		Incidence rate per 1000 person‐years (95% CI)		Risk estimate		Median time to event^a^, years		Excluded individuals due to prior event, *n*	
		pSS^b^	Controls^c^	pSS	Controls	pSS	Controls	Hazard ratio	95% CI	pSS	Controls	pSS	Controls
Myocardial infarction	All	53 (5.6%)	333 (3.7%)	9450	91 958	5.6 (4.3–7.3)	3.6 (3.3–4.0)	1.6	1.2–2.1	8.9	8.5	12	110
	SSA/SSB DP^d^	16 (4.1%)	113 (3.1%)	4152	40 183	3.9 (2.4–6.3)	2.8 (2.3–3.4)	1.4	0.8–2.4	11.7	8.7	3	32
	SSA/SSB SP^e^	17 (6.2%)	86 (3.4%)	2546	24 886	6.7 (4.2–10.7)	3.5 (2.8–4.3)	2.0	1.2–3.4	7.1	8.5	6	43
	SSA/SSB neg.^f^	19 (7.0%)	123 (4.8%)	2625	25 519	7.2 (4.6–11.3)	4.8 (4.0–5.8)	1.5	0.9–2.5	10.3	7.9	3	32
Cerebral infarction	All	34 (3.6%)	278 (3.1%)	9497	92 615	3.6 (2.6–5.0)	3.0 (2.7–3.4)	1.2	0.9–1.7	11.3	8.7	5	85
	SSA/SSB DP	18 (4.6%)	104 (2.8%)	4089	40 252	4.4 (2.8–7.0)	2.6 (2.1–3.1)	1.7	1.0–2.9	13.6	8.1	1	25
	SSA/SSB SP	7 (2.5%)	84 (3.3%)	2601	25 129	2.7 (1.3–5.6)	3.3 (2.7–4.1)	0.8	0.4–1.8	13.1	8.5	3	30
	SSA/SSB neg.	8 (2.9%)	85 (3.3%)	2675	25 803	3.0 (1.5–6.0)	3.3 (2.7–4.1)	0.9	0.4–1.9	9.0	9.7	1	29
Venous thromboembolism	All	50 (5.3%)	238 (2.7%)	9282	92 300	5.4 (4.1–7.1)	2.6 (2.3–2.9)	2.1	1.6–2.9	7.7	8.5	25	126
	SSA/SSB DP	24 (6.3%)	80 (2.2%)	3976	40 262	6.0 (4.0–9.0)	2.0 (1.6–2.5)	3.1	1.9–4.8	5.1	10.4	9	45
	SSA/SSB SP	13 (4.8%)	76 (3.0%)	2580	25 006	5.0 (2.9–8.7)	3.0 (2.4–3.8)	1.7	0.9–3.0	9.7	7.9	7	35
	SSA/SSB neg.	12 (4.5%)	74 (2.9%)	2598	25 656	4.6 (2.6–8.1)	2.9 (2.3–3.6)	1.6	0.9–3.0	9.5	7.6	8	43

The analyses were performed on all primary Sjögren's syndrome patients, and stratified by SSA and SSB autoantibody profile. Individuals with registered events before primary Sjögren's syndrome diagnosis date were excluded. Seventeen primary Sjögren's syndrome patients did not have available records on Ro/SSA and La/SSB antibodies, and are hence not included in subgroup analyses.

CI, confidence interval; SSA, Ro/SSA antibodies; SSB, La/SSB antibodies.

^a^In subjects experiencing the event; ^b^Primary Sjögren's syndrome patients; ^c^General population comparators; ^d^Ro/SSA and La/SSB double‐positive; ^e^Ro/SSA and/or La/SSB single‐positive; ^f^Ro/SSA and La/SSB‐negative.

Stratifying by autoantibody profile, the hazard ratio of MI after Sjögren's syndrome diagnosis was 1.4 (95% CI 0.8–2.5) in SSA/SSB double‐positive patients, 2.0 (95% CI 1.2–3.4) in SSA/SSB single‐positive patients, and 1.5 (95% CI 0.9–2.5) in SSA/SSB‐negative patients (Table 2). When also stratifying by age, both SSA/SSB double and SSA/SSB single‐positive patients had significantly increased hazard ratios of MI after the age of 70 years (Table S2).

### Cerebral infarction

**Figure 2:**
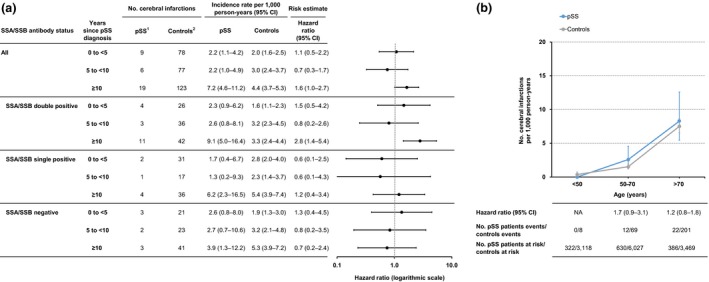
Cerebral infarction in relation to primary Sjögren's syndrome duration and age. (a) Incidence rate and hazard ratio of cerebral infarction in primary Sjögren's syndrome, stratified by years since Sjögren's syndrome diagnosis. Seventeen patients with pSS did not have available records on Ro/SSA and La/SSB antibodies and are not included in subgroup analyses. (b) Age‐specific incidence rate and hazard ratio of cerebral infarction with 95% confidence intervals (CI) in prevalent Sjögren's syndrome, and in population‐based comparators. ^1^Primary Sjögren's syndrome patients, ^2^General population comparators. CI, confidence interval; NA, not applicable.

The incidence rate of cerebral infarction was 3.6 (95% CI 2.6–5.0) per 1000 person‐years in patients with Sjögren's syndrome and 3.0 (95% CI 2.7–3.4) in the comparison cohort, associating with a hazard ratio of 1.2 (95% CI 0.9–1.7) (Table 2). Stratifying based on disease duration, a significantly increased risk was observed in patients after ≥10 years of follow‐up (HR 1.6, 95% CI 1.0–2.7) (Fig. 2a). No cerebral infarction was observed in patients younger than 50 years. The incidence rate of cerebral infarction increased with age, yet with no corresponding increase in observed relative risk in the aggregated group of patients (Fig. 2b, Table S2).

When stratifying by autoantibody profile, SSA/SSB double‐positive patients displayed a significantly increased risk for cerebral infarction (HR 1.7, 95% CI 1.0–2.9), which was not observed in patients single positive or negative for these autoantibodies (Table 2). The increased risk in the SSA/SSB double‐positive group was most prominent after ≥10 years’ disease duration (HR 2.8, 95% CI 1.4–5.4), and in the ages of 50–70 years (HR 2.9, 95% CI 1.3–6.4) (Fig. 2a and Table S2).

### Venous thromboembolism

**Figure 3:**
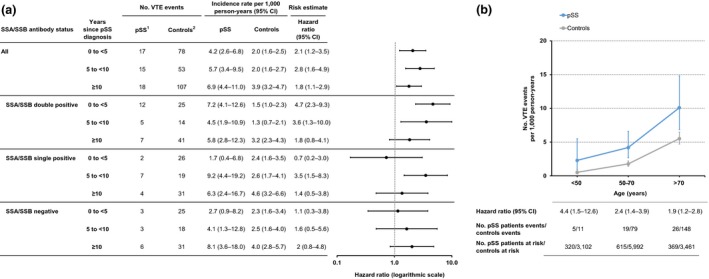
Venous thromboembolism in relation to primary Sjögren's syndrome duration and age. (a) Incidence rate and hazard ratio of venous thromboembolism (VTE) in primary Sjögren's syndrome, stratified by years since Sjögren's syndrome diagnosis. Seventeen patients with Sjögren's syndrome did not have available records on Ro/SSA and La/SSB antibodies and are not included in subgroup analyses. (b) Age‐specific incidence rate and hazard ratio of VTE with 95% confidence intervals (CI) in prevalent Sjögren's syndrome, and in population‐based comparators. ^1^Primary Sjögren's syndrome patients, ^2^General population comparators. CI, confidence interval; NA, not applicable.

Overall, patients with Sjögren's syndrome had a twofold higher risk of VTE (HR 2.1, 95% CI 1.6–2.9) compared to the general population controls, with incidence rates of 5.4 (95% CI 4.1–7.1) and 2.6 (95% CI 2.3–2.9) per 1000 person‐years, respectively (Table 2). An increased risk of VTE was observed already during the first five years after Sjögren's syndrome diagnosis (HR 2.1, 95% CI 1.2–3.5), as well as between 5 to <10 years (HR 2.8, 95% CI 1.6–4.9), and ≥10 years after diagnosis (HR 1.8, 95% CI 1.1–2.9) (Fig. 3a). The incidence rate of VTE in patients with Sjögren's syndrome increased with age, and was consistently higher than that in controls across all ages, with the highest relative risk observed in patients younger than 50 years (HR 4.4, 95% CI 1.5–12.6) (Fig. 3b). Moreover, patients with primary Sjögren's syndrome were significantly more likely to have a history of VTE at the time of diagnosis compared to controls (Table S4).

Stratifying by autoantibody status, the estimated relative risk of VTE after Sjögren's syndrome diagnosis was higher and only significant in SSA/SSB double‐positive patients (HR 3.1, 95% CI 1.9–4.8), compared to SSA/SSB single‐positive (HR 1.7, 95% CI 0.9–4.8) and SSA/SSB‐negative patients (HR 1.6, 95% CI 0.9–3.0) (Table 2). Notably, an increased risk for VTE in SSA/SSB double‐positive patients was particularly evident during the first years after diagnosis (HR 4.7, 95% CI 2.3–9.3) (Fig. 3a). Stratifying by age, the highest risk in SSA/SSB double‐positive patients was observed between 50 and 70 years of age (HR 5.9, 95% CI 3.0–11.4) (Table S2).

Separate analyses of pulmonary embolism and DVT resulted in hazard ratios similar to those of VTE. Similar to VTE, the highest relative risk of pulmonary embolism and DVT was observed in SSA/SSB double‐positive patients (Table S5).

## Discussion

We here report that individuals with primary Sjögren's syndrome have a significantly higher incidence rate of cardiovascular disease in the form of MI, cerebral infarction and VTE. The most striking excess risk was of VTE, for which the aggregated group of patients with Sjögren's syndrome displayed a twofold increased risk compared to the general population. This estimate is in close proximity with that of a recent meta‐analysis reviewing four studies, calculating a pooled risk‐ratio of 2.05 29. Notably, however, by stratifying patients according to autoantibody status, we found that the risk of VTE was in fact substantially higher in patients with SSA and SSB autoantibodies, reaching hazard ratios approaching 5 during the years following diagnosis, while not significant for patients with only one antibody specificity or lacking these autoantibodies. The risk of VTE in patients with primary Sjögren's syndrome has been suggested to relate chronic inflammation, which increases coagulability 30, and is also observed in other systemic autoimmune diseases 21, 28, 29, 31. Moreover, type I IFN, the expression of which can be induced by Ro/SSA and La/SSB autoantibodies, is known to exert multiple adverse effects on the vasculature 11, 12, 32, 33, 34, 35. The degree and character of the systemic inflammation in SSA/SSB double‐positive patients may thus explain the high risk of VTE in this subgroup of patients.

We also observed that Sjögren's syndrome was associated with an increased risk of arterial ischaemic diseases. Overall, the patients had a 1.6‐fold higher relative risk of MI compared to the general population. This estimate is consistent with two previous studies of population‐based cohorts in Sweden and Taiwan, estimating relative risks of 1.6 and 1.2, respectively 19, 20. By contrast, another population‐based study in Taiwan did not note an increased risk 18. This observation may however be explained by the short median follow‐up time of 3.7 years. Indeed, we did not observe an increased risk of MI during the first 5 years following the Sjögren's syndrome diagnosis in our cohort. Of note, no MI events were observed in patients under the age of 50 in our cohort, contrasting to RA and SLE for which increased risk is observed also in younger patients 27, 36.

Cerebral infarction occurred significantly more frequently in patients with Sjögren's syndrome than in controls after 10 or more years from diagnosis. The risk was confined to SSA/SSB double‐positive patients, and in this group reached a HR of 1.6. The observed overall relative risk in our study was similar to the corresponding estimate of 1.3 reported by *Zöller et al*., which is slightly lower than the corresponding risk in RA and SLE 22.

Higher frequencies of subclinical atherosclerosis or endothelial dysfunction have been reported in patients with primary Sjögren's syndrome, as determined by carotid intima‐media thickness, ankle‐brachial index or endothelium‐dependent flow mediated or nitrate‐mediated vasodilation 37, 38, 39, 40. Moreover, disease duration appears to be an important factor in the pathogenesis. *Rachapalli et al*. 41 demonstrated that the ankle‐brachial index was significantly reduced only in patients with a disease duration of more than 10 years. These findings are consistent with our study, in which the relative risk of both MI and cerebral infarction increased with disease duration. In most cases of arterial ischaemic cardiovascular disease, the underlying cause is atherosclerosis, which is widely considered a chronic inflammatory disease 42. Inflammation is increasingly recognized as playing a key role in cardiovascular disease development, and may be the common mechanism underlying the effect of many traditional risk factors 43. Interestingly, Ro/SSA and La/SSB antibodies, which are associated with an activated inflammatory type I IFN system, have been reported to correlate positively with subclinical atherosclerosis and endothelial dysfunction 37, 38, 40. These reports are thus consistent with our findings of higher risks of cerebral arterial events in Ro/SSA‐ and La/SSB‐positive patients. However, intra‐group variations with regards to reaching statistical significance were observed in the analyses. This may partially relate to the limited number of events in each substratum, and calls for caution in interpreting individual findings. Nevertheless, it does not preclude the joint conclusion that the presence of these antibodies associates with an increased risk.

The study has limitations to consider. We were unable to control for lifestyle factors and comorbid disorders potentially influencing the risk of cardiovascular disease as previous studies have suggested that, patients with Sjögren's syndrome have an increased frequency of traditional risk factors associated with cardiovascular disease, including hypertension, hypercholesterolaemia and hypertriglyceridaemia 16, 23, 44, and higher prevalence of subclinical atherosclerosis and endothelial dysfunction 39. However, our matching based on age, sex and geographical region may mitigate confounding effects from lifestyle factors, and adjustment for comorbid disorders has had marginal impact in previous studies 17, 18, 19. The lack of data to account for the presence of antiphospholipid antibodies constitutes another limitation 45, 46. Further, we had no information on underlying treatment, which may potentially influence the risk of cardiovascular disease 47.

The strengths of our study relate to the contextually large size of the cohorts, the long follow‐up and that cardiovascular events were identified using the NPR, which has been confirmed to hold high validity 26. Most importantly though, we included only patients fulfilling AECG criteria 25 and through our clinical characterization could stratify analyses based on Ro/SSA and La/SSB autoantibodies. The presence or absence of these autoantibodies mark two genetically and clinically distinct subgroups, and therefore constitutes an important factor to take into account 48.

Investigations into the mechanisms behind the increased risk of cardiovascular disease in patients with primary Sjögren's syndrome should involve the assessment of both traditional and disease‐specific risk factors. Previous studies of traditional risk factors have mostly not been able to stratify patients according to antibody positivity. A study of the impact of smoking and lifestyle factors using the same cohort of patients with primary Sjögren's syndrome, and hence information on antibody status, is currently underway. Patients positive for SSA/SSB more often present with organ involvement, leukopenia, hypergammaglobulinaemia and high disease activity measured by the European League Against Rheumatism Disease Activity Index (ESSDAI), compared with antibody‐negative patients 49, 50. It is plausible that high disease activity confers an increased risk for cardiovascular disease. While CRP is not a good marker for disease activity in primary Sjögren's syndrome, a prospective study assessing clinical and laboratory manifestations as well as ESSDAI at diagnosis and follow‐up would improve the understanding of disease‐related risk factors. Investigation of the IFN signature, pro‐inflammatory cytokines and biomarkers of endothelial damage as reviewed in *Valim et al*. 39, measured at diagnosis and during the disease course, stratified by SSA/SSB antibodies, would hopefully elucidate some of the immunological disease mechanisms behind the increased risk.

## Conclusion

In this cohort study, we found that patients with primary Sjögren's syndrome have a considerably increased risk of cardiovascular disease of both venous and arterial origin. We further add to the current knowledge by demonstrating that positivity for both Ro/SSA and La/SSB autoantibodies mark the subgroup of patients that carry most of the risk of cerebral infarction and VTE, highlighting the importance of taking patient subgroups into account for correctly defining comorbidity risks. The observations suggest that monitoring and prevention of cardiovascular disease in patients with primary Sjögren's syndrome should be considered, and that Ro/SSA and La/SSB autoantibodies may be used as biomarkers to identify the group of patients with the greater need.

## Conflict of interest statement

The authors declare no competing interests.

## Supporting information


**Table S1.** ICD codes and registers used to identify cardiovascular events.
**Table S2.** Risk of myocardial infarction, cerebral infarction, and venous thromboembolism in prevalent primary Sjögren's syndrome, stratified by SSA and SSB autoantibodies and age.
**Table S3.** Risk of cardiovascular disease after primary Sjögren's syndrome diagnosis, stratified by sex.
**Table S4.** Frequencies of cardiovascular events occurring before primary Sjögren's syndrome diagnosis.
**Table S5.** Risk of pulmonary embolism and deep vein thrombosis events after pSS diagnosis.
