# Supplementary material for: Concomitant Ro/SSA and La/SSB antibodies are biomarkers for the risk of venous thromboembolism and cerebral infarction in primary Sjögren's syndrome
Source: J Intern Med. 2019 Jun 17;286(4):458–68. doi: 10.1111/joim.12941 (PMC6851863; doi:10.1111/joim.12941)
Supplement: Supplementary file 1 — Table S1. ICD codes and registers used to identify cardiovascular events. Table S2. Risk of myocardial infarction, cerebral infarction, and venous thromboembolism in prevalent primary Sjögren's syndrome, stratified by SSA and SSB autoantibodies and age. Table S3. Risk of cardiovascular disease after primary Sjögren's syndrome diagnosis, stratified by sex. Table S4. Frequencies of cardiovascular events occurring before primary Sjögren's syndrome diagnosis. Table S5. Risk of pulmonary embolism and deep vein thrombosis events after pSS diagnosis. [file JOIM-286-458-s001.docx]

**SUPPLEMENTARY TABLES**

| **Supplementary table 1.** ICD codes and registers used to identify cardiovascular events. | | | |
| --- | --- | --- | --- |
| **Outcome variable** | **ICD 10** | **ICD 9** | **Register (diagnosis type)** |
| **Myocardial infarction** | I21 | 410 | Inpatient register (main diagnosis) Cause of death register (main and secondary diagnoses) |
| **Cerebral infarction** | I63 | 434 | Inpatient register (main diagnosis) Cause of death register (main and secondary diagnoses) |
| **Venous thromboembolism** | I26, I80.1-I80.2,  I81, I82.2-I82.9 | 415, 451, 452, 453 | [Pulmonary embolism + deep vein thrombosis] |
| **Pulmonary embolism** | I26 | 415 | Inpatient register (main and secondary diagnoses) Cause of death register (main and secondary diagnoses) |
| **Deep vein thrombosis** | I80.1-I80.2, I81,  I82.2-I82.9 | 451, 452, 453 | Inpatient register (main and secondary diagnoses) Outpatient care register (main and secondary diagnoses) Cause of death register (main and secondary diagnoses) |
|  |  |  |  |

| **Supplementary table 2.** Risk of myocardial infarction, cerebral infarction, and venous thromboembolism in prevalent primary Sjögren’s syndrome, stratified by SSA and SSB autoantibodies and age. | | | | | | | | | | | | | | | | | |
| --- | --- | --- | --- | --- | --- | --- | --- | --- | --- | --- | --- | --- | --- | --- | --- | --- | --- |
|  |  |  | **50 to 70 years** | | | | | | |  | **> 70 years** | | | | | | |
|  |  |  | **No. events (%)** | |  | **Incidence rate per 1,000 person-years (95% CI)** | |  | **Risk estimate** |  | **No. events (%)** | |  | **Incidence rate per 1,000 person-years (95% CI)** | |  | **Risk estimate** |
| **Event** | **SSA/SSB**  **antibody status** |  | **pSS^1^** | **Controls^2^** |  | **pSS** | **Controls** |  | **Hazard ratio**  **(95% CI)** |  | **pSS** | **Controls** |  | **pSS** | **Controls** |  | **Hazard ratio**  **(95% CI)** |
| **Myocardial infarction** | All |  | 14 (2.2%) | 112 (1.9%) |  | 3.0 (1.8-5.1) | 2.5 (2.1-3.0) |  | 1.2 (0.7-2.1) |  | 39 (10.3%) | 218 (6.4%) |  | 15.1 (11.1-20.7) | 8.3 (7.2-9.4) |  | 2.0 (1.4-2.8) |
|  | SSA/SSB DP^3^ |  | 4 (1.6%) | 46 (1.9%) |  | 2.1 (0.8-5.5) | 2.5 (1.8-3.3) |  | 0.8 (0.3-2.3) |  | 12 (9.4%) | 64 (5.6%) |  | 13.1 (7.5-23.1) | 6.8 (5.4-8.7) |  | 2.1 (1.1-4.0) |
|  | SSA/SSB SP^4^ |  | 4 (2.2%) | 27 (1.6%) |  | 3.1 (1.2-8.4) | 2.2 (1.5-3.2) |  | 1.4 (0.5-4.0) |  | 13 (12.3%) | 59 (6.1%) |  | 18.7 (10.9-32.3) | 8.2 (6.4-10.6) |  | 2.6 (1.4-4.8) |
|  | SSA/SSB neg.^5^ |  | 6 (3.1%) | 38 (2.1%) |  | 4.4 (2.0-9.7) | 2.9 (2.1-4.0) |  | 1.5 (0.6-3.6) |  | 13 (9.5%) | 85 (6.8%) |  | 14.4 (8.3-24.7) | 9.3 (7.5-11.5) |  | 1.7 (0.9-3.0) |
| **Cerebral infarction** | All |  | 12 (1.9%) | 69 (1.1%) |  | 2.6 (1.5-4.6) | 1.5 (1.2-2.0) |  | 1.7 (0.9-3.1) |  | 22 (5.7%) | 201 (5.8%) |  | 8.3 (5.5-12.6) | 7.5 (6.6-8.6) |  | 1.2 (0.8-1.8) |
|  | SSA/SSB DP |  | 8 (3.2%) | 27 (1.1%) |  | 4.2 (2.1-8.4) | 1.4 (1.0-2.1) |  | 2.9 (1.3-6.4) |  | 10 (7.8%) | 71 (6.1%) |  | 11.1 (6.0-20.7) | 7.6 (6.0-9.6) |  | 1.5 (0.8-3.0) |
|  | SSA/SSB SP |  | 2 (1.1%) | 23 (1.3%) |  | 1.6 (0.4-6.3) | 1.9 (1.2-2.8) |  | 0.8 (0.2-3.6) |  | 5 (4.6%) | 60 (6.1%) |  | 6.7 (2.8-16.1) | 8.2 (6.4-10.6) |  | 0.8 (0.3-2.1) |
|  | SSA/SSB neg. |  | 2 (1.0%) | 19 (1.0%) |  | 1.4 (0.4-5.7) | 1.4 (0.9-2.2) |  | 1.0 (0.2-4.3) |  | 6 (4.3%) | 65 (5.2%) |  | 6.4 (2.9-14.2) | 7.0 (5.5-8.9) |  | 1.0 (0.4-2.2) |
| **Venous thrombo-embolism** | All |  | 19 (3.1%) | 79 (1.3%) |  | 4.2 (2.7-6.6) | 1.8 (1.4-2.2) |  | 2.4 (1.4-3.9) |  | 26 (7.0%) | 148 (4.3%) |  | 10.1 (6.9-14.8) | 5.5 (4.7-6.5) |  | 1.9 (1.2-2.8) |
|  | SSA/SSB DP |  | 14 (5.8%) | 24 (1.0%) |  | 7.6 (4.5-12.8) | 1.3 (0.9-1.9) |  | 5.9 (3.0-11.4) |  | 8 (6.7%) | 50 (4.3%) |  | 9.1 (4.6-18.2) | 5.3 (4.0-7.0) |  | 1.8 (0.8-3.8) |
|  | SSA/SSB SP |  | 2 (1.1%) | 29 (1.7%) |  | 1.6 (0.4-6.3) | 2.4 (1.7-3.4) |  | 0.7 (0.2-2.8) |  | 9 (8.3%) | 43 (4.4%) |  | 12.2 (6.4-23.5) | 5.8 (4.3-7.9) |  | 2.2 (1.1-4.5) |
|  | SSA/SSB neg. |  | 3 (1.6%) | 26 (1.4%) |  | 2.2 (0.7-6.8) | 2.0 (1.3-2.9) |  | 1.1 (0.3-3.7) |  | 8 (6.0%) | 47 (3.7%) |  | 8.9 (4.5-17.8) | 5.0 (3.8-6.7) |  | 1.8 (0.8-3.8) |
| ^1^primary Sjögren’s syndrome patients, ^2^General population comparators, ^3^Ro/SSA and La/SSB double positive, ^4^Ro/SSA and/or La/SSB single positive, ^5^Ro/SSA and La/SSB negative.  Abbreviations: SSA, Ro/SSA antibodies; SSB, La/SSB antibodies; CI, confidence interval; NA, not applicable.  Seventeen primary Sjögren’s syndrome patients did not have available records on Ro/SSA and La/SSB antibodies, and are hence not included in subgroup analyses. | | | | | | | | | | | | | | | | | |

| **Supplementary table 3.** Risk of cardiovascular disease after primary Sjögren’s syndrome diagnosis, stratified by sex. | | | | | | | | | | | | | | | | |
| --- | --- | --- | --- | --- | --- | --- | --- | --- | --- | --- | --- | --- | --- | --- | --- | --- |
|  |  |  | **No. events (%)** | |  | **Person-years** | |  | **Incidence rate per 1,000 person-years (95% CI)** | |  | **Risk estimate** | |  | **Median time to event^3^, years** | |
| **Event** | **Sex** |  | **pSS^1^** | **Controls^2^** |  | **pSS** | **Controls** |  | **pSS** | **Controls** |  | **Hazard ratio** | **95% CI** |  | **pSS** | **Controls** |
| **Myocardial infarction** | **females** |  | 46 (5.2%) | 286 (3.4%) |  | 8,849 | 86,087 |  | 5.2 (3.9-6.9) | 3.3 (3.0-3.7) |  | 1.6 | (1.2-2.2) |  | 10.0 | 8.4 |
|  | **males** |  | 7 (10.1%) | 47 (7.6%) |  | 601 | 5,871 |  | 11.6 (5.6-24.4) | 8.0 (6.0-10.7) |  | 1.5 | (0.7-3.4) |  | 8.2 | 9.0 |
| **Cerebral infarction** | **females** |  | 32 (3.6%) | 251 (3.0%) |  | 8,861 | 86,485 |  | 3.6 (2.6-5.1) | 2.9 (2.6-3.3) |  | 1.3 | (0.9-1.8) |  | 12.2 | 8.6 |
|  | **males** |  | 2 (2.8%) | 27 (4.3%) |  | 635 | 6,131 |  | 3.1 (0.8-12.6) | 4.4 (3.0-6.4) |  | 0.7 | (0.2-3.1) |  | 5.6 | 8.8 |
| **Venous thromboembolism** | **females** |  | 45 (5.2%) | 216 (2.6%) |  | 8,662 | 86,144 |  | 5.2 (3.9-7.0) | 2.5 (2.2-2.9) |  | 2.1 | (1.5-2.9) |  | 8.1 | 8.3 |
|  | **males** |  | 5 (7.1%) | 22 (3.5%) |  | 620 | 6,156 |  | 8.1 (3.4-19.4) | 3.6 (2.4-5.4) |  | 2.3 | (0.9-6.2) |  | 5.4 | 11.5 |
| ^1^primary Sjögren’s syndrome patients, ^2^General population comparators, ^3^in subjects experiencing the event.  Abbreviations: CI, confidence interval. | | | | | | | | | | | | | | | | |

| **Supplementary table 4.** Frequencies of cardiovascular events occurring before primary Sjögren’s syndrome diagnosis. | | | | | | | |
| --- | --- | --- | --- | --- | --- | --- | --- |
|  |  |  | **Risk estimate** | |  | **Frequency, n (%)** | |
| **Event** | **SSA/SSB antibody status** |  | **Odds ratio** | **95% CI** |  | **pSS^1^** | **Controls^2^** |
| **Myocardial infarction** | All |  | 0.9 | (0.5 - 1.7) |  | 12 (1.3%) | 110 (1.2%) |
|  | SSA/SSB double positive |  | 0.8 | (0.2 - 2.6) |  | 3 (0.8%) | 32 (0.9%) |
|  | SSA/SSB single positive |  | 1.2 | (0.5 - 2.8) |  | 6 (2.2%) | 43 (1.7%) |
|  | SSA/SSB negative |  | 0.8 | (0.3 - 2.8) |  | 3 (1.1%) | 32 (1.2%) |
| **Cerebral infarction** | All |  | 0.5 | (0.2 - 1.3) |  | 5 (0.5%) | 85 (0.9%) |
|  | SSA/SSB double positive |  | 0.4 | (0.0 - 2.8) |  | 1 (0.3%) | 25 (0.7%) |
|  | SSA/SSB single positive |  | 0.8 | (0.2 - 2.8) |  | 3 (1.1%) | 30 (1.2%) |
|  | SSA/SSB negative |  | 0.3 | (0.0 - 2.2) |  | 1 (0.4%) | 29 (1.1%) |
| **Venous thromboembolism** | All |  | 1.9 | (1.2 - 2.9) |  | 25 (2.6%) | 126 (1.4%) |
|  | SSA/SSB double positive |  | 1.9 | (0.9 - 3.9) |  | 9 (2.3%) | 45 (1.2%) |
|  | SSA/SSB single positive |  | 1.8 | (0.8 - 4.2) |  | 7 (2.5%) | 35 (1.3%) |
|  | SSA/SSB negative |  | 1.7 | (0.8 - 3.8) |  | 8 (2.9%) | 43 (1.7%) |
| ^1^primary Sjögren’s syndrome patients, ^2^General population comparators. Abbreviations: SSA, Ro/SSA antibodies; SSB, La/SSB antibodies; CI, confidence interval. Seventeen primary Sjögren’s syndrome patients did not have available records on Ro/SSA and La/SSB antibodies, and are hence not included in subgroup analyses. | | | | | | | |

| **Supplementary table 5.** Risk of pulmonary embolism and deep vein thrombosis events after pSS diagnosis. | | | | | | | | | | | | | | | | |
| --- | --- | --- | --- | --- | --- | --- | --- | --- | --- | --- | --- | --- | --- | --- | --- | --- |
|  |  |  | **No. events (%)** | |  | **Person-years** | |  | **Incidence rate per 1,000 person-years (95% CI)** | |  | **Risk estimate** | |  | **Median time to event^3^, years** | |
| **Event** | **SSA/SSB antibody status** |  | **pSS^1^** | **Controls^2^** |  | **pSS** | **Controls** |  | **pSS** | **Controls** |  | **Hazard ratio** | **95% CI** |  | **pSS** | **Controls** |
| **Pulmonary embolism** | All |  | 25 (2.6%) | 109 (1.2%) |  | 9,523 | 93,528 |  | 2.6 (1.8-3.9) | 1.2 (1.0-1.4) |  | 2.3 | (1.5-3.5) |  | 9.7 | 8.8 |
|  | SSA/SSB double positive |  | 10 (2.6%) | 35 (1.0%) |  | 4,113 | 40,692 |  | 2.4 (1.3-4.5) | 0.9 (0.6-1.2) |  | 2.9 | (1.4-5.8) |  | 5.8 | 11.3 |
|  | SSA/SSB single positive |  | 8 (2.9%) | 36 (1.4%) |  | 2,614 | 25,396 |  | 3.1 (1.5-6.1) | 1.4 (1.0-2.0) |  | 2.2 | (1.0-4.7) |  | 8.5 | 7.9 |
|  | SSA/SSB negative |  | 6 (2.2%) | 35 (1.4%) |  | 2,665 | 26,012 |  | 2.3 (1.0-5.0) | 1.3 (1.0-1.9) |  | 1.7 | (0.7-4.0) |  | 12.3 | 7.8 |
| **Deep vein thrombosis** | All |  | 34 (3.6%) | 160 (1.8%) |  | 9,415 | 92,799 |  | 3.6 (2.6-5.1) | 1.7 (1.5-2.0) |  | 2.1 | (1.5-3.1) |  | 6.7 | 9.1 |
|  | SSA/SSB double positive |  | 19 (4.9%) | 57 (1.6%) |  | 4,052 | 40,442 |  | 4.7 (3.0-7.4) | 1.4 (1.1-1.8) |  | 3.4 | (2.0-5.7) |  | 5.2 | 11.3 |
|  | SSA/SSB single positive |  | 6 (2.2%) | 48 (1.9%) |  | 2,607 | 25,130 |  | 2.3 (1.0-5.1) | 1.9 (1.4-2.5) |  | 1.2 | (0.5-2.8) |  | 8.1 | 8.3 |
|  | SSA/SSB negative |  | 8 (3.0%) | 49 (1.9%) |  | 2,627 | 25,847 |  | 3.0 (1.5-6.1) | 1.9 (1.4-2.5) |  | 1.6 | (0.8-3.4) |  | 6.4 | 7.8 |
| ^1^primary Sjögren’s syndrome patients, ^2^General population comparators, ^3^in subjects experiencing the event. Abbreviations: SSA, Ro/SSA antibodies; SSB, La/SSB antibodies; CI, confidence interval; NA, not applicable. Seventeen primary Sjögren’s syndrome patients did not have available records on Ro/SSA and La/SSB antibodies, and are hence not included in subgroup analyses. | | | | | | | | | | | | | | | | |
